# Resilience enhancement of an urban road network during traffic accidents by optimally dispatching rescue teams

**DOI:** 10.1371/journal.pone.0330824

**Published:** 2025-09-02

**Authors:** Jinqu Chen, Yanni Ju, Shaochuan Zhu, Xiaowei Liu, Xinyue Hu

**Affiliations:** 1 Public Security Department, Fujian Police College, Fuzhou, China; 2 Department of Road Traffic Management, Sichuan Police College, Luzhou, China; 3 School of Transportation and Logistics, Southwest Jiaotong University, Chengdu, China; Universiti Sains Malaysia, MALAYSIA

## Abstract

The efficient dispatch of rescue teams (RTs) during traffic accidents is crucial for the rapid restoration of normal operations in the affected urban road network (URN), thereby enhancing the network’s resilience during such events. However, previous studies focusing on optimizing RT dispatch strategies to enhance URN resilience remain limited. To address this gap, this paper develops a mixed-integer linear programming model aimed at optimizing RT dispatch during traffic accidents. The formulated model is solved using the commercial solver (i.e., CPLEX). Numerical experiments conducted on a hypothetical URN demonstrate that the model generates an optimal dispatch scheme. Compared to baseline strategies, the optimized scheme reduces the total objective function values by 27.36% in small-scale cases and 16.28% in large-scale case, respectively. Furthermore, sensitivity analysis reveal that accident severity and destination locations significantly influence the dispatch scheme design. Finally, the paper discusses the impact of several parameters on the model’s solution, showing that its performance is highly sensitive to several critical factors like RT dispatch costs, the maximum allowable delay time, passenger value of time, and vehicle travel speeds.

## 1. Introduction

With the rapid acceleration of the urbanization in China, vehicle ownership continues to increase. Official statistics reveal that the number of registered cars grew from 279 million in 2015–417 million in 2022 [1]. While this growth has made travel more convenient, it has also led to increased traffic congestion and a corresponding rise in accidents. For instance, the number of traffic accidents in China increased from 187,781 in 2015–256,409 in 2022. These accidents not only disrupt the normal operation of the urban road network (URN) but also result in serious casualties. Rescue teams (RTs), such as traffic police, who are responsible for incident management and investigation, play a crucial role in restoring URN functionality after accidents [2]. However, RT availability is often constrained in practice, creating an urgent need for optimal dispatch strategies when demand exceeds available resources. Therefore, an effective RT dispatch scheme is essential for minimizing network disruption time and enhancing URN resilience during traffic accidents.

Multiple metrics, including vulnerability, robustness, and resilience, have been adopted to quantify the ability of URNs to cope with disruptions such as natural disasters and traffic accidents [3,4]. Among these metrics, resilience emerges as the most comprehensive indicator for assessing the capability of a URN to respond to disruptions [5,6]. Resilience, stemmed from the Latin “resiliro”, means to spring back or bounce back [7]. The concept of resilience was first applied to urban transportation systems in 2006 to evaluate their adaptive capacity during disruptions [8]. After nearly two decades of development, URN resilience research comprises both resilience assessment and enhancement [9,10]. Studies on URN resilience assessment typically evaluate the capability of a URN to deal with disruptions according to the negative effects caused by them. For instance, Vivek and Conner [11] assessed the resilience of Boston’s URN by analyzing the cascading effects of targeted infrastructure attacks. Ma et al. [12] established a multidimensional framework that incorporates resilience loss, robustness, redundancy, resourcefulness, and recovery to evaluate the URN resilience in flood scenarios. Liu et al. [13] assessed the resilience of a URN based on four metrics (i.e., robustness, recovery, rapidity, and performance loss) by considering the impact of cascading failure. Generally, the duration affected by disruptions and the performance deviation caused by disruptions are widely applied resilience metrics in current studies [9].

Based on the resilience assessment results, researchers have developed various strategies to enhance the resilience of URNs [14]. Some measures, such as network structure optimization [15], perimeter control strategies [16], and restoration sequence optimization [17], have been applied to enhance URN resilience. Zhang et al. [18] implied that rapidly recovering normal operations of the affected URN significantly helps to improve its ability to respond to disruptions, emphasizing that quick restoration is a crucial aspect of improving URN resilience. Several approaches, including the repair sequence optimization of failed components [19] and traffic signal optimization [20], have been employed in current studies to quickly recover normal operations of the affected URN. For example, Amini et al. [21] quantitatively validated how dynamic traffic control measures can improve URN resilience during congestion events.

Traffic accidents are the most frequent disruptions affecting the normal operation of a URN in its daily operation. Scholarly investigations into URN traffic accidents have systematically addressed three critical dimensions: (1) traffic accident prediction [22], (2) influencing factors analysis [23], and (3) real-time operational management during accidents [24]. Unlike studies focusing on accident prevention primarily aim to avoid traffic accidents, research on traffic management during accidents emphasizes the development of measures to restore the normal operation of affected networks. Several approaches have been applied to manage traffic accidents, including traffic control system design and vehicle rerouting [25]. For instance, Qi et al. [26] designed a traffic signal-based emergency control policy to help prevent large-scale congestion induced by traffic accidents. Wang et al. [27] developed a dynamic adaptive vehicle rerouting strategy to mitigate the negative impacts of traffic congestion on URN operations. Although various strategies have been developed from different perspectives to manage traffic accidents in URNs, all researchers highlight the importance of effective traffic accident management in improving network resilience during such events [28].

Although researchers have conducted extensive work to enhance the resilience of URNs during traffic accidents, strategies for enhancing URN resilience through the optimal dispatch of RTs remain limited. However, RTs play an important role in handling traffic accidents, and their effectiveness in restoring the normal operation of affected URNs cannot be overlooked. To address this gap, a mixed-integer linear programming (MILP) model is developed to optimally dispatch RTs during traffic accidents, thereby enhancing the resilience of URNs under such conditions. A solution approach utilizing the commercial solver CPLEX is employed to solve the formulated model. Numerical experiments conducted on a hypothetical URN are performed to validate the model’s effectiveness. The main contributions of this paper are twofold. First, a MILP model is developed to optimize RT dispatch schemes during accidents. Second, various traffic accident scenarios are studied, and practical implementations are provided to enhance the resilience of a URN under traffic accidents.

The remainder of this paper is organized as follows. The next section presents the formulation of the model for optimal RT dispatch during traffic accidents. The experiments conducted in the Numerical experiments section are used to verify the effectiveness of the proposed model and solution approach. Finally, the main conclusions and future research directions are presented in the Conclusions section.

## 2. Methodology

### 2.1. Problem statement and notations

#### 2.1.1. Problem statement.

Properly organizing accident response tasks for RTs during traffic accidents not only reduces costs but also accelerates the restoration of affected URNs. In China, RTs such as traffic police squadrons are the primary agencies responsible for handling traffic accidents within their patrol duty areas. However, these squadrons are not authorized to manage traffic accidents occurring outside their patrol areas. Consequently, only the patrol duty area of a specifc RT is studied herein. Moreover, only on-duty RTs can handle traffic accidents.

**Fig 1:**
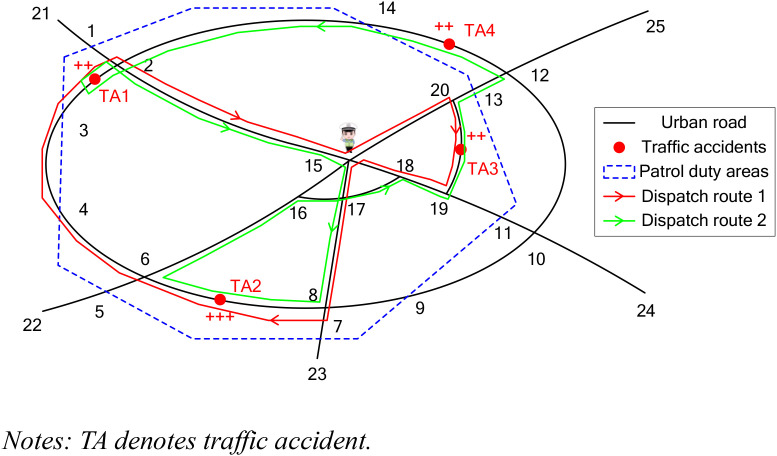
Problem statement.

In this paper, the URN within the patrol duty area of an RT is defined as $G(N_{\text{1}}\text{,}N_{\text{2}}\text{,}E)$, where *N*_1_, *N*_2_, and *E* represent the sets of road intersections and endpoints, intersections between roads and the patrol duty area, and road segments, respectively. The hypothetical URN shown in Fig 1 is used to illustrate the impact of different RT dispatch strategies on network restoration during traffic accidents. As depicted in Fig 1, the network contains 25 nodes (including 16 road intersections and endpoints, and 9 intersections between roads and the patrol duty area). Four traffic accidents exist in the network, with traffic accidents 1–3 occurring within the patrol duty area. The “+” symbols near traffic accidents denote their severity, where a greater number of “+” symbols indicates that the corresponding traffic accident is more serious.

Assuming that the available RT is located at node 15 when traffic accidents 1–4 occur, the command center should dispatch the team at node 15 to handle accidents 1–3 to restore the normal operation of the affected URN within the patrol duty area. Typically, the command center dispatches available RTs based on the severity and locations of traffic accidents [29]. As shown in Fig 1, there are two possible dispatch routes. The route indicated by the red line shows that the team first deals with traffic accident 2 and finally handles traffic accident 3, while the route indicated by the green line shows that the RT first handles traffic accident 2 and finally deals with traffic accident 1. It is found that the route marked by the red line is more optimal than that marked by the green line, as it has a shorter distance.

As discussed above, the RT dispatch scheme is influenced by several factors, including the available RTs, the URN structure, and the severity and locations of traffic accidents. In simple cases with a limited number of available RTs and traffic accidents, the optimal RT dispatch scheme can be readily obtained using an enumeration approach. However, the complex network structure and numerous available RTs make it difficult to obtain an optimal dispatch scheme using the enumeration approach. Hence, a MILP model is developed to efficiently solve this problem. The model aims to minimize the cost of dispatching RTs, shorten the duration affected by traffic accidents, and maximize the rescue fairness across different traffic accidents. Before presenting the model, several assumptions are made:

A1. RTs travel between different locations using the shortest path.
A2. Accidents are resolved immediately upon the arrival of RTs, meaning that the time required for accident management is negligible.
A3. The approximate severity of each accident is known prior to the design of the dispatch scheme.

#### 2.1.2. Notations.

The notations used in this paper are tabulated in Table 1.

**Table 1:** Notations used in this paper.

Notations	Descriptions
**Sets**	
*A*	Set of traffic accidents.
*K*	Set of available RTs.
*O* ^*k*^	Set of origin locations of the RT *k*.
*D* ^*k*^	Set of destination locations of the RT *k*.
**Parameters**	
*s*_*i*_	Approximate severity of the traffic accident *i*.
$t_{ij}^{k}$	Travel time from the scene of the traffic accident *i* to *j* of the RT *k*.
ν	Passenger value of time (VOT).
$\rho^{k}$	Cost for the RT *k* to complete the task.
ξ	Penalty coefficient.
$\varphi_{1}$, and $\varphi_{2}$	Model weights.
γ	Maximum allowed delay time.
**Variables**	
$x_{i}^{k}$	$x_{i}^{k}\in \{0,1\}$, $x_{i}^{k}=1$ if the RT *k* is dispatched to handle the traffic accident *i*, and 0 otherwise.
$y_{ij}^{k}$	$y_{ij}^{k}\in \{0,1\}$, $y_{ij}^{k}=1$ if the RT *k* handles the accident *j* immediately after it deals with the accident *i*, and 0 otherwise.
$a_{i,o}^{k}$	$a_{i,o}^{k}\in \{0,1\}$, $a_{i,o}^{k}=1$ if the RT *k* departs from the origin location *o* to deal with the traffic accident *i*, and 0 otherwise.
$z_{i,d}^{k}$	$z_{i,d}^{k}\in \{0,1\}$, $z_{i,d}^{k}=1$ if the RT *k* returns to the destination location *d* after it handle the traffic accident *i*, and 0 otherwise.
$p_{i}$	$p_{i}\in R^{+}$, penalty time of the traffic accident *i*.
$w_{i}^{k}$	$w_{i}^{k}\in R^{+}$, the arrival time of the RT *k* at the scene of the accident *i*.
$w_{i}$	$w_{i}\in R^{+}$, complete time of the traffic accident *i*.

### 2.2. Model formulation

#### 2.2.1. Objective function.

When normal operations of a URN are disrupted by traffic accidents, the command center typically aims to rapidly restore operations quickly while minimizing dispatch costs. The costs of dispatching RTs during traffic accidents comprise personnel costs, vehicle operating costs, and penalty costs. Therefore, the first component (*F*_1_) of the objective function focuses to minimize RT dispatch costs, which is calculated using Equation (1).


$$\begin{array}{cc} F_{1}= & \min\sum_{k\in K}\sum_{i\in A}\rho^{k}\cdot x_{i}^{k}+\nu \cdot \sum_{k\in K}\sum_{i\in A}\sum_{j\in A}t_{ij}^{k}\cdot y_{ij}^{k}+\nu \cdot \sum_{k\in K}\sum_{o\in O^{k}}\sum_{i\in A}t_{oi}^{k}\cdot a_{i,o}^{k}+\nu \cdot \sum_{k\in K}\sum_{d\in D^{k}}\sum_{i\in A}t_{id}^{k}\cdot z_{i,d}^{k}\\ & +\nu \cdot \sum_{i\in A}\xi \cdot p_{i} \end{array}$$ (1)

The second component (*F*_2_) of the objective function aims to minimize the disruption duration caused by traffic accidents. In this study, the disruption duration is defined as the maximum time required for RTs to arrive at all accident scenes. That is:


$$F_{2}=\text{max}\;w_{i},\forall i\in A$$ (2)

*F*_2_ is subsequently reformulated as Equations (3) and (4) to linearize its nonlinear components.


$$F_{2}=g$$ (3)

$$g\geq \text{max}\;w_{i},\forall i\in A$$ (4)

If the proposed model primarily focuses on optimizing rescue time and cost, it may cause excessive delays in rescue times for certain accidents, resulting in significant disparities in the overall rescue times. To minimize this rescue time unfairness, the third component ($F_{\text{3}}$) of the objective function is designed to maximize rescue fairness across different traffic accidents, as quantified in Equation (5).


$$F_{3}=\text{min}\left(\text{max}\left(w_{i}-w_{j}\right)\right),i\neq j,\forall i,j\in A$$ (5)

To address the nonlinear component, $F_{3}$ is transformed as below.


$$F_{3}=\text{min}\;\varrho$$ (6)

$$\varrho \geq w_{i}-w_{j},i\neq j,\forall i,j\in A$$ (7)

As illustrated above, $F_{\text{1}}$ optimizes the RT dispatch scheme by minimizing total costs. $F_{\text{2}}$ focuses on reducing the total disruption duration, while $F_{\text{3}}$ minimizes the range between the earliest and latest rescue times. These sub-objective functions have distinct optimization goals. Therefore, weighting coefficients $\varphi_{1}$ and $\varphi_{2}$ are introduced to reflect the relative importance of $F_{\text{1}}$, $F_{\text{2}}$, and $F_{\text{3}}$ in the proposed model. Higher values of $\varphi_{1}$ and $\varphi_{2}$ indicate a greater emphasis on minimizing both the disruption duration and the range between rescue times. In this study, $\varphi_{1}=\varphi_{2}=\text{1}$, with the impact of weight values to be discussed subsequently. To sum up, the overall objective function *F* of the proposed model is:


$$\text{min}\;F=\;F_{\text{1}}+\varphi_{1}\cdot \nu \cdot F_{\text{2}}+\varphi_{2}\cdot \nu \cdot F_{\text{3}}$$ (8)

#### 2.2.2. Constraints.

For each traffic accident *i*, it must be serviced by an RT exactly once. That is:


$$\begin{array}{c} \sum_{k\in K}x_{i}^{k}=\text{1},\forall i\in A \end{array}$$ (9)

The arrival time of an RT at a traffic accident scene is determined by its departure time and travel duration. Therefore, the arrival time of RT *k* at accident *i* is ensured by Constraint (10). Furthermore, a coupling constraint (i.e., Constraint (11)) links variables $w_{i}^{k}$ and $x_{i}^{k}$. Finally, Constraint (12) guarantees the completion time for accident *i*.


$$\begin{array}{c} w_{j}^{k}\geq \sum_{i\in A}\left(w_{i}^{k}+t_{ij}^{k}\right)\cdot y_{ij}^{k},\forall j\in A,k\in K \end{array}$$ (10)

$$w_{i}^{k}\geq T_{1}\cdot x_{i}^{k},\forall i\in A,k\in K$$ (11)

$$\begin{array}{c} w_{i}\leq \sum_{k\in K}w_{i}^{k},\forall i\in A \end{array}$$ (12)

where $T_{\text{1}}$ is the minimal vehicle travel time among a given node pair.

To handle the nonlinear part in Constraint (10), it is further reformulated as Constraints (13) – (17).


$$\begin{array}{c} w_{j}^{k}\geq \sum_{i\in A}\tau_{ij}^{k}+\sum_{i\in A}t_{ij}^{k}\cdot y_{ij}^{k},\forall j\in A,k\in K \end{array}$$ (13)

$$\tau_{ij}^{k}\leq T_{2}\cdot y_{ij}^{k}\text{,}\forall i\text{,}j\in A\text{,}k\in K$$ (14)

$$\tau_{ij}^{k}\leq w_{i}^{k}\text{,}\forall i\text{,}j\in A\text{,}k\in K$$ (15)

$$\tau_{ij}^{k}\geq w_{i}^{k}-T_{2}\cdot \left(1-y_{ij}^{k}\right)\text{,}\forall i\text{,}j\in A\text{,}k\in K$$ (16)

$$\tau_{ij}^{k}\geq 0\text{,}\forall i\text{,}j\in A\text{,}k\in K$$ (17)

where $\tau_{ij}^{k}$ denotes an intermediate variable. $T_{\text{2}}$ represents a large constant.

In actual operations, command centers typically prioritize available RTs to address higher-severity accidents, indicating that these critical incidents require immediate response:


$$s_{i}\geq \left(y_{ij}^{k}-1\right)\cdot M_{1}+s_{j},\forall i,j\in A,k\in K$$ (18)

Constraint (18) is equivalent to Constraints (19) – (20) after executing the linearization operation.


$$s_{i}\geq \left(y_{ij}^{k}-1\right)\cdot M_{1}+s_{j},\forall i,j\in A,k\in K$$ (19)

$$s_{i}\leq y_{ij}^{k}\cdot M_{1}+s_{j}-\epsilon_{1},\forall i,j\in A,k\in K$$ (20)

where $M_{\text{1}}$ and $\epsilon_{\text{1}}$ are constants. In this paper, $M_{\text{1}}=\text{1}$ and $\epsilon_{\text{1}}=\text{0}.\text{01}$.

Constraints (21) and (22) limit the first and last traffic accidents to be serviced by each RT.


$$a_{i}^{k}$$ (21)

$$a_{i}^{k}$$ (22)

where $a_{i}^{k}$ and $z_{i}^{k}$ are binary variables. If RT *k* handles traffic accident *i* first, then $a_{i}^{k}=\text{1}$; and $a_{i}^{k}=\text{0}$ otherwise. Similarly, $z_{i}^{k}=\text{1}$ implies that RT *k* handles the accident *i* last, while $z_{i}^{k}=\text{0}$ denotes that *k* does not handle accident *i* last. $\varsigma_{o}^{k}$ and $\vartheta_{d}^{k}$ denote 0–1 variables. $\varsigma_{o}^{k}=\text{1}$ denotes RT *k* departs from origin location *o*, while $\varsigma_{o}^{k}=\text{0}$ implies *k* does not depart from *o*. If RT *k* finally ends at destination location *d*, then $\vartheta_{d}^{k}=\text{1}$; and $\vartheta_{d}^{k}=\text{0}$ otherwise.

The linearization formulations of Constraints (21) and (22) are presented in Constraints (23) – (25), and Constraints (26) – (28), respectively.


$$a_{i,o}^{k}\leq a_{i}^{k},\forall i\in A,k\in K,o\in O^{k}$$ (23)

$$a_{i,o}^{k}\leq \varsigma_{o}^{k},\forall i\in A,k\in K,o\in O^{k}$$ (24)

$$a_{i,o}^{k}\geq a_{i}^{k}+\varsigma_{o}^{k}-\text{1},\forall i\in A,k\in K,o\in O^{k}$$ (25)

$$z_{i\text{,}d}^{k}\leq z_{i}^{k},\forall i\in A,k\in K,d\in D^{k}$$ (26)

$$z_{i\text{,}d}^{k}\leq \vartheta_{d}^{k},\forall i\in A,k\in K,d\in D^{k}$$ (27)

$$z_{i\text{,}d}^{k}\geq z_{i}^{k}+\vartheta_{d}^{k}-\text{1},\forall i\in A,k\in K,d\in D^{k}$$ (28)

The values of $a_{i}^{k}$ and $z_{i}^{k}$ are determined by $w_{i}^{k}$ based on RT arrival times at designated accident locations.


$$a_{i}^{k}$$ (29)

$$a_{i}^{k}$$ (30)

After linearization, Constraints (29)-(30) are equivalently expressed as Constraints (31)-(33) and Constraints (34)-(36), respectively.


$$w_{i}^{k}\leq \left(1-a_{i}^{k}\right)\cdot M_{2}+G^{k},\forall i\in A,k\in K$$ (31)

$$w_{i}^{k}\geq -a_{i}^{k}\cdot M_{2}+G^{k}+\epsilon_{2},\forall i\in A,k\in K$$ (32)

$$G^{k}\leq \text{min}\;w_{i}^{k},\forall i\in A,k\in K$$ (33)

$$w_{i}^{k}\geq \left(z_{i}^{k}-1\right)\cdot M_{2}+H^{k},\forall i\in A,k\in K$$ (34)

$$w_{i}^{k}\leq z_{i}^{k}\cdot M_{2}+H^{k}-\epsilon_{2},\forall i\in A,k\in K$$ (35)

$$H^{k}\geq \text{max}\;w_{i}^{k},\forall i\in A,k\in K$$ (36)

where $M_{\text{2}}$ and $\epsilon_{\text{2}}$ are large constants, respectively. $M_{\text{2}}=\text{100,000}$ and $\epsilon_{\text{2}}=\text{1}$ herein. $G^{k}$ and $H^{k}$ are intermediate variables.

According to the traffic accident investigation regulations in China [30], RTs are required to arrive at any accident scene within 15 minutes. Hence, the delay time $d_{i}$ for accident *i* is calculated as $\text{max}\left(\text{0,}w_{i}-\gamma \right)$. $d_{i}$ is equivalent to Constraints (37) to (38).


$$d_{i}\geq \text{0},\forall i\in A$$ (37)

$$d_{i}\geq w_{i}-\gamma ,\forall i\in A$$ (38)

To sum up, the proposed model is formulated as a MILP model below.


minF (39)

*subject to* Constraints (4), (7), (9), (11) – (17), (19) – (20), (23) – (28), (31) – (38).

To obtain a solution rapidly based on the proposed model, an efficient solution algorithm is required [31]. The proposed model is a MILP model, which can be efficiently solved by commercial solvers like CPLEX and Gurobi. Therefore, in this paper, by employing CPLEX and the Yalmip library (https://yalmip.github.io/), the proposed model is solved by MATLAB on a windows PC.

## 3. Numerical experiments

### 3.1. Numerical setting

**Fig 2:**
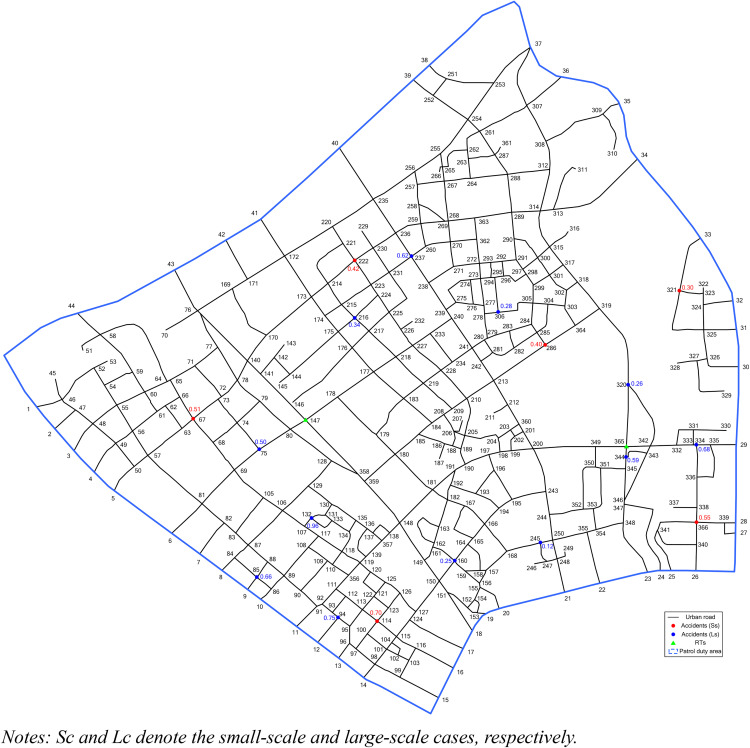
Hypothetical URN.

In this study, numerical experiments based on a hypothetical URN are conducted to validate the effectiveness of the proposed model. The patrol duty area of an RT is delineated by the blue dotted line in Fig 2. As shown in Fig 2, the hypothetical URN comprises 366 nodes (including 322 urban road intersections and endpoints, and 44 intersections between roads and the patrol duty area) and 314 road segments.

Assuming that the road grade in Fig 2 is uniform and that the average vehicle travel speed is 50 km/h according to the design specification, the travel times between nodes are calculated based on distance. According to Hakkert and Mahalel [32], traffic accidents occur more frequently at road intersections. Therefore, this study focuses on intersection accidents, though the method remains applicable to segment-based accidents. Traffic accidents and available RTs are displayed by circles and triangles in Fig 2, respectively. Moreover, the decimal value adjacent to each accident marker (circle) indicates its approximate severity level. For example, the red circle at intersection 67 with the notation “0.51” represents an accident with approximately 0.51 severity at that location.

This study examines two distinct traffic accident scenarios. The first scenario represents a small-scale case with 6 traffic accidents, in which the accident at node 114 shows the highest severity. The second scenario represents a large-scale case comprising 12 traffic accidents, with the most severe incident occurring at node 132. Two RTs positioned at nodes 147 and 365 are available to address these accidents. Upon completing all accident responses, the RTs may return to any of the designated nodes: 55, 124, 147, 173, or 365.

Finally, the additional parameters used in this study are summarized in Table 2. The VOT for passengers is calculated based on per capita income and average working hours, which is set as 60.43 RMB per hour herein [33]. Given that on-duty RT availability is limited (typically only one team is operational in real-world scenarios), dispatching a second RT incurs significantly higher costs. Due to data limitations, RT dispatch costs are assumed herein. The cost for dispatching the first RT is set as 1 RMB each task, while that for dispatching the second RT is set as 20 RMB each task. These values imply that the cost for dispatching the second RT is 20 times higher than that for dispatching the first RT. The impact of the cost for dispatching different RTs will be discussed later. Based on the work of Viscus [34], the penalty coefficient is set as 100. According to [30], the maximum allowable delay time is set as 900 s.

**Table 2:** Other parameters used in the model.

Parameter	Value	Parameter	Value
ν	60.43 RMB/h	ξ	100
ρ	[1, 20]	γ	900 s

### 3.2. Result analysis

The optimal RT dispatch schemes for both small- and large-scale cases are obtained using the proposed model. Table 3 presents the computational time, objective value, and optimal dispatch schemes for these cases. The solution times are 11.88 s for the small-scale case and 3105.40 s for the large-scale case, demonstrating the efficiency of the CPLEX-based solution approach in solving the small-scale case. In Table 3, dispatch schemes are represented by node sequences connected by dashes. For example, the scheme “RT1: 147-114-67-222-173” denotes that RT1 first handles the accident at node 114 and finally addresses the accident at node 222. After completing all assigned tasks, the team returns to node 173. For the small-scale case, the total objective value is RMB 122.67, with the first, second, and third sub-objective values being RMB 99.41, 861.48 s, and 523.8 s, respectively. The optimal scheme for the small-scale case indicates that two RTs are arranged to handle 6 traffic accidents and both teams return to node 173 upon task completion.

**Table 3:** Computational time, objective value, and the optimal RT dispatch scheme.

Case	CT (s)	OV (RMB)	Scheme
Small-scale	11.88	122.67	Team 1: 147-114-67-222-173, Team 2: 365-366-286-321-173.
Large-scale	3105.40	3,543.94	Team 1: 147-132-85-75-216-306-320-160-245-124, Team 2: 365-94-334-237-344-124.

Notes: CT and OV represent the computational time and objective value, respectively.

To further validate the proposed model, the optimal scheme shown in Table 3 is compared with the conventional scheme, which is commonly used in actual operations. In the conventional scheme, the task for each RT is randomly assigned by the command center, and available RTs are dispatched based on the accident severity. That is, accidents with higher severity are prioritized. The objective values obtained under the conventional scheme are RMB 156.23 and RMB 4120.84 for the small- and large-scale cases, respectively. These results demonstrate that the scheme generated by the proposed model is more optimal than the conventional scheme. Specifically, the optimal scheme reduces the objective value by 27.36% and 16.28% for the small- and large-scale cases, respectively.

As illustrated in the previous subsection, two RTs are available to handle the assumed traffic accidents. To evaluate the impact of the number of available RTs on the resilience enhancement effectiveness of the proposed model, Table 4 presents the model’s objective values under different numbers of teams. In this table, RT3 is posintiond at node 147 with a dispatch cost of 20 RMB per task. The model achieves the best objective value for the small-scale case with two available RTs. However, in the large-scale case, the objective value continues to decrease as the number of available RTs increases. These results indicate that appropriately increasing the number of available RTs (i.e., deploying more RTs for duty) is essential in areas where multiple traffic accidents are likely to occur simultaneously. Specifically, based on historical data, the command center can use the proposed model to determine the optimal number of available RTs for a given patrol duty area.

**Table 4:** Impact of the number of available RTs.

NoT	Small-scale case				Large-scale case			
	*F* (RMB)	*F*_1_ (RMB)	*F*_2_ (s)	*F*_3_ (s)	*F* (RMB)	*F*_1_ (RMB)	*F*_2_ (s)	*F*_3_ (s)
1	3,648.57	3,631.38	1,753.20	1415.52	22,820.79	22,723.89	3,024.22	2748.02
2	122.67	99.41	861.48	523.80	3,543.94	3,493.05	1,653.91	1377.72
3	124.18	100.75	866.74	529.06	836.51	806.81	1,022.62	716.42

Notes: NoT denotes the number of available RTs.

As shown in Constraints (18) to (20), the formulated model prioritizes resolving traffic accidents with greater severities. To assess the impact of accident severity on the design of dispatch scheme, Table 5 compares the objective values for the small-scale case with and without consideration of severity. The results indicate that the objective value without considering severity is lower than when severity is considered. This occurs because RT dispatch follows the shortest path among accident scenes when severity is ignored. However, in practice, command centers typically prioritize dispatching available RTs to more severe accidents first, as these often result in greater economic losses and casualties. Therefore, the severity of traffic accidents significantly influences the design of RT dispatch schemes and cannot be overlooked.

**Table 5:** Objective values considering and without considering the impact of the severity.

Considering severity?	*F* (RMB)	*F*_1_ (RMB)	*F*_2_ (s)	*F*_3_ (s)
Yes	122.67	99.41	861.48	523.80
No	99.18	75.66	783.29	617.69

In the Numerical setting subsection, it is assumed that RTs can return to five nodes (i.e., nodes 55, 124, 147, 173, and 365) after completing their assigned tasks. To evaluate the impact of destination locations on the model’s objective value, the RT dispatch scheme that requires returning to the origin locations (i.e., nodes 147 and 365 for RTs 1 and 2, respectively) is analyzed. Table 6 presents a comparison of objective values between schemes that require returning to origin locations and those that do not. The results show that schemes requiring return to origin locations consistently yield higher objective values than those without this requirement. This finding demonstrates that dispatch costs increase when RTs must return to their origin locations, confirming that destination location significantly impacts the design of dispatchs.

**Table 6:** Impact of the need of back to the departure location.

Case	Small-scale case		Large-scale case	
	NBO	DNBO	NBO	DNBO
*F* (RMB)	122.67	129.78	3,543.94	3,546.23

Notes: NBO and DNBO are RT needs and does not need to back to departure location.

To further evaluate the effectiveness of the proposed solution approach, the model is also solved by using a genetic algorithm. Table 7 presents the computational times and objective values obtained by different solution approaches. The results show that although the genetic algorithm requires less computational time than the proposed CPLEX-based solution approach, it yields higher objective values. Furthermore, the genetic algorithm fails to produce a solution for the large-scale case. These findings demonstrate that our CPLEX-based solution approach can effectively solve the formulated MILP model.

**Table 7:** Comparison of different solution algorithms.

Algorithms	Computational time (s)		Objective value (RMB)	
	Small-scale	Large-scale	Small-scale	Large-scale
CPLEX	11.88	3105.40	122.67	3,543.94
Genetic algorithm	0.80	0.31	548.71	No solution

**Fig 3:**
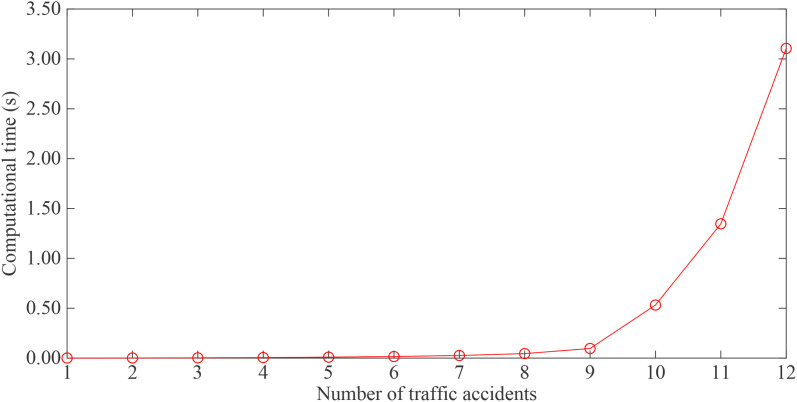
Solution efficiency of the proposed approach.

Fig 3 shows the model’s computational time under different numbers of traffic accidents, indicating a positive exponential relationship between computational time and the number of accidents. The computational time increases from 0.99 s to 3105.40 s as the number of traffic accidents rises from 1 to 12. This trend demonstrates that the computational efficiency of the proposed solution approach decreases with the number of accidetnes increases. However, in real-world scenarios, the average number of simultaneous traffic accidents in urban areas is typically below 5. For example, Chengdu recorded 1,324 traffic accidents in 2023 [35], which corresponds to a daily average of 3.62 accidents. Therefore, the proposed solution method remains practical for the design of actual RT dispatch schemes. Nonetheless, developing more efficient solution approaches represents an important direction for future research.

### 3.3. Discussions

**Fig 4:**
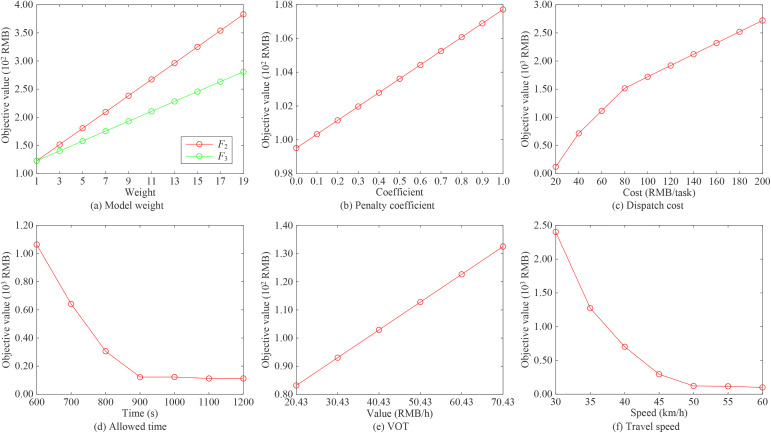
Impact of parameters on the model.

This subsection conducts sensitivity analysis to investigate how various parameters affect the resilience enhancement effectiveness of the proposed model. Using the small-scale case as an example, Fig 4 illustrates the impact of different parameters on the model’s objective value.

As shown in Fig 4a, there is a positive correlation between the model’s objective value and weights $\varphi_{1}$ and $\varphi_{2}$. The fitting equation between the objective value (*F*) and weight ($\varphi_{1}$) is $F=\text{14}.\text{46}\times \varphi_{1}+\text{108}.\text{20}$ (with a goodness of fit of 1.0), while the equation for $\varphi_{2}$ is $F=\text{8}.\text{79}\times \varphi_{2}+\text{113}.\text{90}$ (with a goodness of fit of 1.0). These results indicate that the objective value increases by an average of 14.46 RMB and 8.79 RMB, respectively, for each unit increase in $\varphi_{1}$ and $\varphi_{2}$. This demonstrates that the model weights significantly influence the design of dispatch schemes. Fig 4b illustrates the effect of the penalty coefficient on the objective value. The objective value shows minimal variation with increasing penalty coefficients. Morover, further analysis implies that the objective value remains constant when the penalty coefficient exceeds 50. This occurs because all traffic accidents can be promptly addressed in the small-scale case.

The impact of the cost for dispatching the second RT on the objective value is shown in Fig 4c. The correlation coefficient between the cost and the objective value is 0.978, indicating a strong positive relationship between them. Furthermore, the growth rate of the objective value decreases as the dispatch cost continues to rise. This finding highlights the importance of maintaining sufficient RTs in patrol areas with a high probability of occurring traffic accidents, as it not only ensures rapid restoration of URN operations but also optimizes dispatch costs. Fig 4d illustrates the relationship between the objective value and the maximum allowable delay time. With the increase in the delay time, the objective value initially decreases and then stabilizes. The reason is that the total time required for two RTs to complete the task is 861.48 s, which is below the 900 s. Thus, the maximum allowable delay time only influences the model’s objective value when it is less than 900 s. To mitigate the negative effects of traffic accidents on URN operations, the command center should strategically deploy available RTs to ensure prompt recovery.

As shown in Fig 4e, a strong positive correlation exists between the model’s objective value and the VOT. When the VOT increases from 20.43 RMB/h to 70.43 RMB/h, the objective value rises from 83.17 RMB to 132.54 RMB, representing an increase of 37.25%. Therefore, when traffic accidents affect URNs in areas with high VOT, the command center should promptly design effective RT dispatch schemes to minimize negative impacts. Fig 4f shows that the model’s objective value decreases as vehicle travel speed increases. This finding indicates that dispatch costs for RTs handling traffic accidents can be effectively reduced by improving vehicle speeds. Thus, RTs assigned to accident investigation should utilize emergency lanes to increase their travel speed and enhance response efficiency.

## 4. Conclusions

Effective dispatch of RTs plays a vital role in restoring normal operations of affected URNs following traffic accidents, thereby enhancing network resilience during such accidents. This paper develops a MILP model to optimize the dispatch of RTs during traffic accidents. A CPLEX-based solution approach is employed to solve the developed model. Numerical experiments conducted on a hypothetical URN validate the model’s effectiveness. The key findings of this study are summarized below.

First, the proposed model and solution approach can effectively obtain an optimal RT dispatch scheme during traffic accidents. Compared with the basic scheme that is widely applied in actual operations, the optimal scheme reduces objective values by 27.36% and 16.28% for small- and large-scale cases, respectively. Second, the availability of RTs significantly impacts the model’s solution effectiveness. The command center can use the formulated model to determine the optimal number of available RTs in a specified patrol duty area based on historical data. Moreover, the influences of accident severity and destination locations on the RT dispatch scheme design cannot be ignored. Third, the solution approach developed in this paper provides more efficient and optimal solutions than those obtained using a genetic algorithm. Moreover, a positive exponential relationship exists between the model’s computational time and the number of traffic accidents. Finally, the analysis of various parameters on the model’s objective value reveals that it is significantly impacted by the cost of dispatching a second RT, the maximum allowable delay time, the VOT, and vehicle travel speed.

Despite promising results are obtained in this study, several limitations exist. First, although traffic accident severity is incorporated into the model’s constraints, the impact of its uncertainty on the design of dispatch schemes is not included due to data limitations. Second, the model does not account for uncertainties in vehicle travel time. In practice, despite emergency lane access, rescue vehicle travel time may vary significantly due to complex traffic conditions, particularly during peak hours. Third, the dispatch costs for different RTs are assumed due to a lack of empirical data. The above shortcomings will be addressed in future. Future research will develop a robust optimization model for the design of RT dispatch schemes that incorporates uncertainties in both accident severity and vehicle travel time. Accurate data on the costs for dispatching different RTs will be determined by carrying out a survey. Moreover, more realistic traffic accident scenarios will be considered in the proposed model by relaxing the model assumptions.

## Supporting information

S1 File Supplementary material. (RAR)
